# Myocardial Infarction With Non-obstructive Coronary Arteries: An Updated Overview of Pathophysiology, Diagnosis, and Management

**DOI:** 10.7759/cureus.23602

**Published:** 2022-03-29

**Authors:** Arshan Khan, Abdelilah Lahmar, Maria Riasat, Moiz Ehtesham, Haris Asif, Warisha Khan, Muhammad Haseeb, Hetal Boricha

**Affiliations:** 1 Internal Medicine, Ascension St. John Hospital, Detroit, USA; 2 Medicine, Faculty of Medicine and Pharmacy/Mohammed VI University Hospital, Oujda, MAR; 3 Internal Medicine, Icahn School of Medicine Mount Sinai Beth Israel, New York, USA; 4 Internal Medicine, Albany Medical Center, Albany, USA; 5 Internal Medicine, Woodhull Medical Center, New York, USA; 6 Internal Medicine, Faisalabad Medical University, Faisalabad, PAK; 7 Internal Medicine, Bahria International Hospital, Lahore, PAK; 8 Internal Medicine, Jinnah Hospital Lahore, Lahore, PAK; 9 Internal Medicine, University of Pittsburgh Medical Center (UPMC) McKeesport Hospital, Pittsburgh, USA

**Keywords:** myocardial infarction type ii, coronary plaque disruption, coronary artery dissection, coronary artery spasm, minoca

## Abstract

Myocardial infarction with non-obstructive coronary arteries (MINOCA) refers to acute myocardial infarction with normal or near-normal coronary arteries. The MINOCA is a heterogeneous group of conditions, and possible etiologies are coronary artery spasm, spontaneous coronary artery dissection, coronary thromboembolism, coronary plaque disruption, coronary microvascular dysfunction, supply and demand mismatch. It is more common in young adults, with women having a higher chance of getting MINOCA than men. Considering MINOCA as a clinically dynamic working diagnostic that needs further investigation rather than a “true” diagnosis is proposed. Optical coherence tomography (OCT), intravenous ultrasound (IVUS), cardiac MRI may be required to stratify the underlying mechanism. Due to the lack of evidence-based literature and prospective randomized controlled studies, therapeutic management is limited. Consequently, the strategy is patient-specific. The prognosis of MINOCA patients remains unclear and depends upon the underlying etiology. This article aims to review the literature about various aspects of MINOCA, including pathophysiology, diagnosis, prognosis, and treatment.

## Introduction and background

Most myocardial infarction (MI) cases are caused by rupture of atherosclerotic plaque with subsequent thrombotic occlusion [1-2]. Plaques are generated by intimal thickening, lipid deposition, and macrophage infiltration in the early stages of atherosclerosis; gradually, this atheroma plaque protrudes into the arterial lumen, leading to the formation of arterial stenosis. However, positive remodeling keeps the vascular lumen dilated by remodeling the outer diameter [1]. When this compensatory mechanism is exceeded, the vessel lumen narrows, and severe stenosis causes angina pectoris [1]. Of note, in up to one-third of patients with symptoms suggestive of the acute coronary syndrome (ACS) and elevated troponin levels and/or ischemic ST-segment changes, coronary angiography reveals no obstructive atherosclerosis, implying that functional changes in the epicardial arteries and/or coronary microcirculation could be the pathogenetic cause [1-4]. This later clinical entity is known as myocardial infarction (MI) with nonobstructive coronary arteries (MINOCA) [4].

The MINOCA patients have evidence of myocardial infarction with nonobstructive coronary arteries on angiography (≤50% stenosis) in the absence of overt cause of MI, such as cardiac trauma or injury, and has been reported in 5% to 6% of individuals with acute myocardial infarction (AMI) [2-4]. In patients with MINOCA, female sex and younger age are more common than in individuals with AMI and obstructive coronary artery disease (CAD) [2]. The etiologies of MINOCA include coronary artery spasm, spontaneous coronary artery dissection, plaque disruption or erosion, and microvascular dysfunction [1-4]. Optimal management depends mainly on the underlying pathophysiological mechanism [1-4]. The underlying cause influences the prognosis of the patients.

## Review

Epidemiology

MINOCA was first documented more than 75 years ago on the autopsy of a patient with MI, who was found to have myocardial necrosis in the absence of significant coronary artery disease [5-6]. MINOCA prevalence ranges between 5 and 25% of all MIs [4].

MINOCA is more common in women, and patients are often younger than those with acute myocardial infarction associated with obstructive coronary artery disease (AMI-CAD) [2]. MINOCA is also more common in black or Pacific and Hispanic ethnicity [7-8]. In the large systematic review, the average age of patients with MINOCA was 58 years, compared with 61 years in patients with AMI-CAD. The percentages of hypertension, diabetes mellitus, dyslipidemia, active smokers, ST-segment elevated myocardial infarction (STEMI), and those with a family history of coronary artery disease (CAD) are less frequent in MINOCA patients [2,9].

Etiologies and pathophysiology

The pathophysiology of MINOCA is poorly understood, and it can be divided into the following four categories: epicardial coronary processes, demand and supply mismatch, coronary microvascular dysfunction, and unknown mechanism [10].

Epicardial coronary processes causing MINOCA

The epicardial coronary vessels processes causing MINOCA include coronary artery spasm, spontaneous coronary artery dissection (SCAD), plaque disruption, and in situ thrombosis [10].

Coronary artery spasm

Coronary artery spasm is vasoconstriction of the epicardial coronary artery resulting in insufficient blood flow to the heart [2].

Coronary artery spasm is called focal spasm when it involves one segment of the epicardial artery and multifocal when it involves two or more segments of the same vessel [11-12]. Coronary vasospasm is a common cause of MINOCA. In one study, coronary vasospasm was present in 46% of patients with MINOCA who underwent provocative testing, and incidence was higher in Asian than in Caucasian patients [13-14]. The pathophysiology of coronary artery spasm involves vascular smooth muscle hyperreactivity to endogenous or exogenous stimuli (such as spasms induced by cocaine), imbalance of vagal and sympathetic tone, and endothelial dysfunction [11-12]. The spasm of an epicardial coronary artery is usually brief and causes transient ischemia, and in some cases, the prolonged spasm leads to persistent ischemia, which can cause MINOCA [10].

Spontaneous coronary artery dissection

SCAD is a nontraumatic dissection of an epicardial coronary artery. In most cases, SCAD is generally associated with >50% stenosis and, therefore, is an uncommon cause of MINOCA [15]. SCAD is more common in women compared to men [16]. The SCAD leads to intramural hematoma formation and intimal disruption, leading to coronary artery obstruction [17]. The chief presenting symptom is chest pain. The causes of SCAD are still unclear but probably include emotional or physical stress, use of stimulant or illicit drugs, and changes in the intima-media composition due to hormones and pregnancy [18]. Fibromuscular dysplasia is frequently associated with SCAD [18-19]. Other associations of SCAD are collagen vascular disorders (Marfan syndrome, Ehlers-Danlos syndrome, and Alport syndrome) and inflammatory disorders (systemic lupus erythematosus, celiac disease, sarcoidosis, and inflammatory bowel disease) [19-21]. The true incidence of SCAD is controversial because many events can be missed or misdiagnosed [17]. The incidence ranges from 0.07% to 1.1%, and it is a predominantly disease of women [21-23]. SCAD has been reported to account for one quarter to one-third of MIs in patients younger than 50 [18,24].

Plaque disruption

Plaque disruption is a common cause of MINOCA [25]. The term plaque disruption includes plaque rupture or erosion. Plaque rupture is the disruption of the fibrous cap resulting in communication between coronary lumen and plaque cavity. In contrast, plaque erosion is intraluminal thrombus formation with intact intima without signs of rupture [2]. The main mechanisms of MINOCA in patients with plaque disruption are thrombus formation, which can cause AMI via distal embolization, complete transient occlusion of coronary vessels with subsequent spontaneous thrombolysis, and coronary spasm [2,10].

Angiography is of limited use in the diagnosis of plaque disruption. A definite diagnosis can only be made using optical coherence tomography (OCT) and intravascular ultrasound (IVUS) [10]. In one study, plaque disruption was identified by IVUS in 38% of women with MINOCA.

Demand and supply mismatch

Demand and supply mismatch may lead to MINOCA. MI due to supply and demand mismatch is known as type II MI [10]. Approximately 50% of patients with type II MI do not have significant CAD, and they can be classified as MINOCA [10]. The common causes of demand and supply mismatch are hypotension, tachyarrhythmia, and hypoxia [2,10]. The diagnosis of type II MI is made in an appropriate clinical setting and the absence of imaging or clinical evidence to support a different diagnosis [2].

Coronary microvascular dysfunction

Coronary microvascular dysfunction (CMD) is a common cause of MINOCA. CMD is defined as a clinical angina syndrome with ischemic electrocardiogram changes without obstructive CAD and epicardial spasm [26]. CMD was previously known as Cardiac X syndrome. CMD is more prevalent in women, smokers, diabetics, and hypertensive patients [2]. It affects coronary arteries less than 500 micrometers. The pathophysiology of MINOCA in CMD is impaired vasodilation due to smooth muscle and endothelial dysfunction, which hampers the blood flow regulation to meet myocardial oxygen demand [2,10,26]. CMD can be seen in 30-50% of patients with stable ischemic chest pain and nonobstructive CAD [2,10].

Unknown etiologies

In many patients with MINOCA, the underlying cause remains unknown even after extensive workup [10]. It emphasizes that the pathophysiology of MINOCA is still poorly understood, and more research is needed in this area.

Diagnosis and clinical presentation

Patients with MINOCA have symptoms of cardiac ischemia and elevated cardiac troponins similar to those who have acute myocardial infarction with obstructed coronary arteries [2]. On the electrocardiogram (ECG), they usually have non-ST segment elevation myocardial infarction (NSTEMI). It is important to note that the clinical presentation of MINOCA patients varies due to the many causes of the illness [27-28].

**Figure 1:**
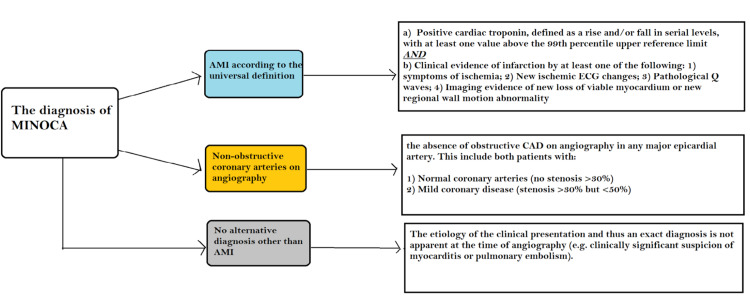
Diagnostic criteria of MINOCA Adapted from reference [32] MINOCA: Myocardial infarction with non-obstructive coronary arteries; CAD: Coronary artery disease; AMI: Acute myocardial infarction.

NSTEMI-MINOCA presentations make up a significant proportion of all MINOCA cases. According to studies, patients with NSTEMI-MINOCA appeared more frequently than STEMI-MINOCA [2, 29-30]. Angiographic criteria include the absence of obstructive disease on angiography (i.e., no coronary artery stenosis ≥50%) in any major epicardial vessel. Although the list is not exhaustive, the absence of alternate diagnoses for the clinical presentation, such as sepsis, myocarditis, and pulmonary embolism, is also a key element in diagnosing MINOCA [2,31]. Figure 1 shows the details of the diagnostic criteria of MINOCA.

When there is no evident cause for increased troponin at presentation, MINOCA should be considered a working diagnosis. As a result, the first step in the evaluation is to rule out non-ischemic causes of an increased troponin level [33]. The European Society of Cardiology (ESC) working group established a diagnostic protocol that comprised a complete history, physical examination, laboratory tests, imaging, and invasive procedures to uncover the underlying cause of MINOCA. This holistic strategy should aid physicians in better managing MINOCA in patients [15]. The diagnosis of MINOCA should be considered a “work in progress” according to the 2017 European Society of Cardiology Working Group on Cardiovascular Pharmacotherapy position paper, which suggests that thorough diagnostic investigations should be performed on every MINOCA patient to determine the causative mechanisms to identify [15]. A working diagnosis of MINOCA is considered if coronary angiography during a suspected AMI reveals non-obstructive coronary arteries and there is no evident reason for the clinical presentation/elevated troponins, such as infection, pulmonary embolism, or myocarditis. Because the prognosis and management differ depending on the etiology, every attempt should be taken to identify the precise etiology.

Treatment

Patients with MINOCA present a significant treatment challenge. Treatment is individually tailored to the underlying physiopathology of each MINOCA subtype. Table 1 discusses the treatment options. The use of renin-angiotensin system blockers and statins has been shown to be beneficial in reducing mortality in patients with MINOCA [34-36]. In an observational study of MINOCA patients enrolled in the SWEDEHEART registry, recruiting 9466 patients with MINOCA, to investigate the associations between treatment with statins, renin-angiotensin system blockers, β-blockers, dual antiplatelet therapy, and long-term cardiovascular events; the use of antiplatelet agents and beta-blockers did not result in a significant reduction in major cardiac events [37]. There is an ongoing randomized MINOCA BAT trial, which is evaluating beta-blockers. Treatment of MINOCA is based on little evidence, and randomized, controlled trials are required to develop a systematic treatment approach [34].

**Table 1:** Summary of the diagnosis and management of MINOCA, according to the underlying physiopathology Adapted from [34, 35] MINOCA: Myocardial infarction with non-obstructive coronary arteries; OCT: Optical coherence tomography; SCAD: Spontaneous coronary artery dissection; IVUS: Intravenous ultrasound; TTE: Transthoracic echocardiogram; TEE: Transesophageal echocardiogram; AMI: Acute myocardial infarction.

Cause	Mechanism	Diagnosis	Treatment
Epicardial Causes	Coronary artery spasm	Coronary vasospasm provocation test with ergonovine and acetylcholine	Calcium channel blockers as first-line therapy or long-acting nitrates in refractory cases
	Spontaneous coronary artery dissection	OCT is the gold standard for SCAD imaging and diagnosis	Conservative treatment (beta-blocker and single antiplatelet therapy) for TIMI II or greater flow. Immediate percutaneous intervention (PCI) for hemodynamically unstable patient or patient with TIMI flow 0 or I
	Coronary plaque disruption	MRI, IVUS, and OCT. MRI imaging can be helpful in diagnosing the problem and guiding care to avoid future problems. However, OCT offers the highest resolution imaging	Aspirin, a platelet P2Y12 receptor blocker, a beta-blocker (in the presence of left ventricular dysfunction), and a statin
	Coronary thromboembolism	TTE, TEE, or bubble contrast echocardiography and coronary angiography. Thrombophilia screening with factor V Leiden, prothrombin 20210A, factor VIII, proteins C and S, antithrombin, lupus anticoagulant, and antiphospholipid antibodies should all be included in the work-up	The treatment is individualized. Percutaneous or surgical closure is required in cases with atrial septal defects. Antiplatelet or anticoagulation medication may be considered for the prevention of coronary embolism with a left-sided etiology
Microvascular cause	Coronary microvascular dysfunction	Microvascular dysfunction is identified by the presence of slow coronary flow on coronary angiography. Cardiac magnetic resonance imaging (MRI) can demonstrate a microvascular blockage. Positron emission tomography, myocardial perfusion imaging, and coronary computed tomography angiography can also be used	Sublingual nitroglycerin, conventional antianginal therapy (beta-blockers and calcium channel blockers)
Myocardial infarction type II	Mismatch in oxygen supply in relation to myocardial metabolic demand	Detection of a rise and/or fall of cardiac troponin values with at least one value above the 99th percentile of the upper reference limit - Evidence of an imbalance between myocardial oxygen supply and demand, requiring at least one of the following features: 1) symptoms of AMI; 2) new ischemic ECG changes; 3) pathological Q waves; 4) imaging evidence of new loss of viable myocardium or new regional wall motion abnormality in a pattern consistent with ischemia	Treatment of the underlying condition
MINOCA of unknown etiology	Unknown	Intravascular imaging	Aspirin, statins, calcium channel blocker

Prognosis

Due to the differences in the pathophysiological signaling pathways, it is difficult to compare the prognosis of MINOCA and MI associated with CAD patients (MI-CAD). The prognosis of MINOCA is closely linked to the etiology of the disease, which should be actively explored [38]. Even in the absence of severe coronary artery obstruction, cardiovascular magnetic resonance imaging diagnosis of acute myocardial infarction and age were significant predictors of major adverse cardiac events (MACE) [39]. Additionally, different studies had diverging results regarding short-term and long-term outcomes. MINOCA patients had a better long-term prognosis during long-term clinical follow-up than MI-CAD [40]. In one large sample study, MINOCA patients had a higher 30 days mortality rate than MI-CAD patients [41].

According to another study, there was no significant difference in MACE rates between MINOCA and patients with obstructive coronary artery disease. The MINOCA group had a lower cardiovascular death rate (p=0.03), but all-cause mortality was not significant [42].

## Conclusions

MINOCA is a recent entity with many distinct etiologies and different pathophysiologies. The clinician should be aware of this syndrome and various etiologies so that patient can be diagnosed and treated accordingly. The diagnosis should be considered in patients presenting with AMI, no significant coronary artery obstruction (i.e., < 50%), and no other explanation for myocardial injury without ischemia. The diagnosis tools used to diagnose MINOCA include coronary angiography, OCT, IVUS, MRI, transthoracic echocardiogram (TTE), and transesophageal echocardiogram (TEE). Identification of the underlying cause is of utmost importance, as prognosis and treatment vary depending on the underlying cause. The use of statins and ACE/ARBS has been shown to reduce mortality in MINOCA patients. On the other hand, aspirin, clopidogrel, and beta-blocker have not demonstrated any clear mortality benefits. Further randomized clinical trials are warranted to evaluate the therapeutic approach for MINOCA patients.
